# Longer treatment duration with SLIT leads to higher patient satisfaction and clinical improvement. Outcomes of the SAMITES study

**DOI:** 10.1186/2045-7022-3-S2-P12

**Published:** 2013-07-16

**Authors:** Enric Marti, Carlos Sanchez-Salguero, Enrique Scorza, Mario Alberto Garcia

**Affiliations:** 1 Clinica Internacional de Medicina Avanzada (CIMA), Allergy Service, Barcelona, Spain; 2 Hospital Virgen de las Montañas, Allergy Service, Cadiz, Spain; 3 Stallergenes Iberica, Medical Department, Barcelona, Spain

## Background

Sublingual immunotherapy (SLIT) is considered as a valid alternative in treatment of patients suffering house dust mite (HDM) allergic rhinitis. No evaluation of the patients' satisfaction on treatment and its relationship with clinical improvement and compliance was previously described.

## Methods

An observational cross-sectional study was carried out to compare patients' satisfaction after 4-6 months (group A) or 9-12 months (group B) of SLIT treatment by the use of a validated Spanish satisfaction questionnaire (ESPIA) consisting of 16 items on a 5-point Likert scale (scale 16-80, higher score indicating more satisfaction). Secondary objectives were to investigate relation between satisfaction and both clinical improvement and compliance. Patients were classified in terms of compliance into <25%; 25-50%, 51-75%; >76% group.

## Results

Data from 232 patients (162 in A and 70 in B) were collected. In group A, 72% of them had persistent and 96% moderate-severe rhinitis before starting immunotherapy. Similarly in group B, 68% of them had persistent and 97% moderate-severe rhinitis. Treatment duration was 5.5**±**1.7 months in A and 12.3**±**2.5 months in B. Median total satisfaction was 60 and 73 points in A and B respectively. In those patients reporting compliance > 76% (n=191) higher median values of satisfaction (ESPIA score) were found in group B (P<0.0001). Patients changing from persistent to intermittent rhinitis between start of SLIT and current situation were 49% and 60% in A and B respectively. Concerning severity according to ARIA guidelines, patients changing from moderate-severe to mild were 43% and 72% in A and B respectively (group effect, p=0.0003). In those patients reporting clinical improvement in terms of rhinitis frequency (n=108) higher median values of satisfaction were found in group B (P=0.0007) and similarly, patients reporting clinical improvement in terms of rhinitis severity (n=151), higher median values of satisfaction were found in group B (P<0.0001).

## Conclusions

Almost 50% of the patients treated 5.5 months experienced an improvement of their rhinitis in terms of frequency (49%) and severity (43%), this percentage is higher in patients treated 12.3 months 60% and 72% respectively following ARIA classification.The SAMITES study demonstrates a clear relationship between patients' satisfaction with SLIT for HDM allergic rhinitis with the duration of treatment, compliance and clinical improvement.

